# Impulsive Choices Emerge When the Anterior Cingulate Cortex Fails to Encode Deliberative Strategies

**DOI:** 10.1523/ENEURO.0379-24.2024

**Published:** 2024-11-15

**Authors:** Shelby M. White, Mitchell D. Morningstar, Emanuela De Falco, David N. Linsenbardt, Baofeng Ma, Macedonia A. Parks, Cristine L. Czachowski, Christopher C. Lapish

**Affiliations:** ^1^Psychology Department, Indiana University-Purdue University, Indianapolis, Indiana 46202; ^2^Neuroscience, EPFL Center for Neuroprosthetics, Lausanne 1015, Switzerland; ^3^Department of Neurosciences, University of New Mexico, Albuquerque, New Mexico 87131; ^4^Department of Anatomy, Cell Biology, and Physiology, Stark Neuroscience Institute, Indianapolis, Indiana 46202

**Keywords:** decision-making, delay discounting, electrophysiology, impulsivity, optogenetics, prefrontal cortex

## Abstract

Impulsive individuals excessively discount the value of delayed rewards, and this is thought to reflect deficits in brain regions critical for impulse control such as the anterior cingulate cortex (ACC). Delay discounting (DD) is an established measure of cognitive impulsivity, referring to the devaluation of rewards delayed in time. This study used male Wistar rats performing a DD task to test the hypothesis that neural activity states in ACC ensembles encode strategies that guide decision-making. Optogenetic silencing of ACC neurons exclusively increased impulsive choices at the 8 s delay by increasing the number of consecutive low-value, immediate choices. In contrast to shorter delays where animals preferred the delay option, no immediate or delay preference was detected at 8 s. These data suggest that ACC was critical for decisions requiring more deliberation between choice options. To address the role of ACC in this process, large-scale multiple single-unit recordings were performed and revealed that 4 and 8 s delays were associated with procedural versus deliberative neural encoding mechanisms, respectively. The 4 and 8 s delay differed in encoding of strategy corresponding to immediate and delay run termination. Specifically, neural ensemble states at 4 s were relatively stable throughout the choice but exhibited temporal evolution in state space during the choice epoch that resembled ramping during the 8 s delay. Collectively, these findings indicate that ensemble states in ACC facilitate strategies that guide decision-making, and impulsivity increases with disruptions of deliberative encoding mechanisms.

## Significance Statement

Investigating the neural basis of impulsivity has broad implications, from understanding decision-making to treating psychopathology. The role of the anterior cingulate cortex (ACC) in mediating impulsivity during delay discounting (DD) remains unclear; however, increasing evidence suggests a role in guiding decision-making strategies. Here we report that disrupting ACC activity when rats are required to use a deliberative decision-making strategy increases impulsivity. These findings provide evidence that ACC is uniquely important for guiding deliberative decision-making during DD.

## Introduction

Delay discounting (DD) is a phenomenon that describes the tendency for temporally delayed rewards to lose value, and the extent of devaluation is an indication of cognitive impulsivity (Wittmann and Paulus, 2008). DD is an evolutionarily conserved behavior that has been observed in every species examined to date (Vanderveldt and Green, 2017). Importantly, extreme DD (i.e., high impulsivity) is a characteristic feature of several psychiatric disorders (see Peterson et al., 2015 for review). Therefore, identifying neural activity patterns that underlie decisions during DD has broad implications, from understanding decision-making to treating psychopathology.

Using strategies to guide decision-making can reduce impulsivity (Linsenbardt et al., 2017; Sweis et al., 2018). Strategy refers to a set of rules used to guide behavior during decision-making and can be identified by differences in choice patterns, such as alternating between choice options (Rich and Shapiro, 2009; Bissonette and Roesch, 2015; Hasz and Redish, 2018). Decisions influenced by DD are hypothesized to arise from the interaction of procedural and deliberative decision-making strategies, each of which have their own computational processes and neural correlates (Van Der Meer et al., 2012; McLaughlin and Redish, 2023). In sum, we define deliberative decision-making here as an effortful process to compare the value of available options, whereas procedural decision-making employs rules or strategies that limit the need for deliberation.

At both the psychological and neural level, deliberation has been conceptualized as a process where evidence for the best outcome is accumulated and terminates once a decision is made (Gold and Shadlen, 2007; Papale et al., 2012). The accumulation of information has been hypothesized to be facilitated by “ramping” activity in single neurons, where firing rates progressively increase up to the decision point (Maoz et al., 2013; Jahans-Price et al., 2014; Waskom and Kiani, 2018). In a DD task, when deliberating between immediate and delayed options, the value of the delayed option needs to be considered at the time in the future when it will be received (J. Peters et al., 2010; Kurth-Nelson et al., 2012; Papale et al., 2012). Deliberation therefore requires cognitive effort, unlike procedural decision-making which is guided by pre-established evidence (for reviews, see Van Der Meer et al., 2012; McLaughlin et al., 2021). In tasks that have a repeated, consistent trial structure, a procedural decision-making strategy is advantageous as it requires less cognitive effort by limiting the need to evaluate the options during each trial (Brouwer et al., 2010; McLaughlin and Redish, 2023). Therefore, determining how strategies impact impulsivity can provide insight into the mechanisms that underlie decision-making during DD.

Impaired function of the PFC is thought to contribute to high impulsivity, and, supporting this view, rats with altered anterior cingulate cortex (ACC) function and neurochemistry are both highly impulsive and lack behavioral correlates of strategy (Engleman et al., 2006; Beckwith and Czachowski, 2014; Linsenbardt and Lapish, 2015; Linsenbardt et al., 2017, 2019; De Falco et al., 2021; Timme et al., 2022; McLaughlin and Redish, 2023). However, of the studies that have examined ACC function during DD, mixed effects are observed (Cardinal et al., 2001; Churchwell et al., 2009; Loos et al., 2010; Feja and Koch, 2014; Sonntag et al., 2014; McLaughlin and Redish, 2023). These discrepancies may be attributable to differences in task parameters (e.g., delay duration) or the hypotheses about the role of the ACC in DD that guide the study.

Several studies have assessed the role of the ACC in value encoding as well as more abstract processes during DD. While value representations are observed in ACC, this region likely contributes more abstract processes during decision-making in DD (Laskowski et al., 2016; Sackett et al., 2019). In line with this view, neural correlates of switching between procedural or deliberative decision-making, initiation of deliberation, and the need to change strategy have been observed in ACC (G. J. Peters et al., 2013; Schuck et al., 2015; Powell and Redish, 2016; Schmidt et al., 2019; McLaughlin and Redish, 2023). Together, these data motivate the need to identify computations performed by ACC during DD to understand its role in impulsive decision-making.

In this study, we investigate the hypothesis that neural activity in ACC plays a critical role in limiting impulsive decisions via strategy selection when cognitive effort is required. To test this hypothesis, optogenetics and multiple single-unit neural recordings were accomplished in male rats performing an adjusting amount DD task.

## Materials and Methods

### Animals

Male Wistar rats were purchased from Envigo for optogenetic inhibition of ACC (*n* = 8) and awake-behaving electrophysiology in ACC (*n* = 10). Animals were acclimated for 3 d following arrival to the vivarium. A 12 h reverse light/dark cycle with lights off at 7:00 A.M. was utilized. Following acclimation, animals were single housed and given at least a week prior to testing. Animals were at least 70 d of age prior to testing and had *ad lib* access to food and water prior to food restriction/habituation. Animals were food restricted to 85% of their starting free-feeding weight and maintained under this condition throughout all experiments except immediately prior to and up to 7 d after surgery. All procedures were approved by the IUPUI School of Science Institutional Animal Care and Use Committee and were in accordance with the National Institutes of Health Guidelines for the Care and Use of Laboratory Animals.

### Operant apparatuses

Eight standard one-compartment operant boxes (20.3 cm × 15.9 cm × 21.3 cm; Med Associates) inside of sound-attenuating chambers (ENV-018M; Med Associates) were used for both optogenetic and electrophysiology experiments in the habituation and shaping protocols. Each box contained left and right retractable levers on one wall, left and right stimulus lights positioned immediately above each lever, and an easily accessible pellet hopper positioned between these left and right devices. The opposite wall contained a house light and a tone generator (2,900 Hz) on the topmost position. One custom-built operant box (21.6 cm × 25.7 cm × 52.0 cm) was used to accommodate all electrophysiological experiments. Dimensions, stimuli (including house and cue lights), and retractable levers were all positioned to replicate the conditions of the standard operant boxes as closely as possible. The floor bars of the custom-built box were made of wood polls rather than metal and all metal components of the box were covered in a powder coating to reduce artifacts. In addition, two of the eight standard operant chambers were modified for optogenetic inhibition (see below, Stimulation and recording equipment, for additional information).

### Behavioral procedures

Following single housing, animals were handled daily for a week. Animals were then habituated to the operant chambers and completed pretraining in the same manner described in previous work from our group (Linsenbardt et al., 2017) prior to beginning the DD task. The within-session adjusting amount DD procedure was a modified version of the procedure performed by Linsenbardt et al. (2017), which was adapted from Oberlin and Grahame (2009). Before beginning the DD task, the immediate and delay levers were assigned for each animal during shaping. Choosing the delay lever always resulted in the delivery of six sucrose pellets following a delay (0, 1, 2, 4, 8, or 16 s), while the immediate (adjusting) lever dispenses 0–6 pellets with no delay. The number of pellets dispensed by the immediate lever (*i*-value) on the first trial of each session was three pellets. Each delayed choice increased and each immediate choice decreased *i*-value by one pellet on the subsequent trial.

**Figure 1.**
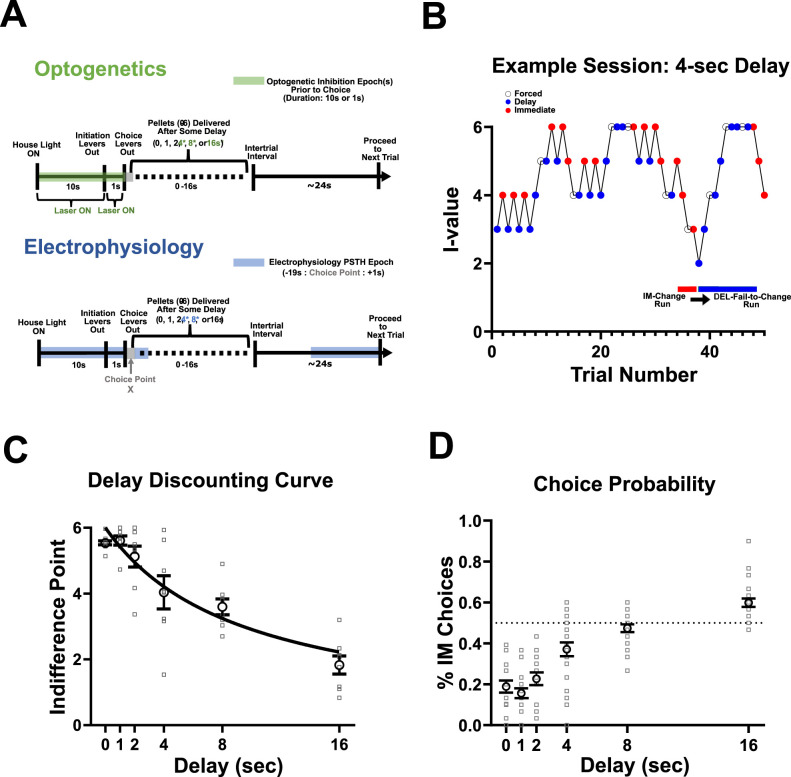
Description of optogenetics and electrophysiology experiments and evidence of discounting. ***A***, Description of a single trial during DD depicting optogenetic inhibition of ACC (***A***, top) and epochs selected for analysis in awake-behaving recordings from ACC (***A***, bottom). The green highlighted portion of the trial depicts the points during a single trial where laser was turned ON (***A***, top) while the blue highlighted region (***A***, bottom) depicts the portion of the trial analyzed for the electrophysiology experiment. ***B***, Example single-session depicting how an animal makes choices during the 4 s delay. Choice trials are depicted in red (immediate choices) and blue (delay choices) while forced trials are shown in white. An example of IM-Change and DEL-Fail-to-Change run is depicted by the red and blue horizontal lines, respectively, at the bottom. *i*-value refers to the number of pellets dispensed by the adjusting (Immediate) lever on a given trial. ***C***, Mazur hyperbolic DD curve fit to indifference points across delays (0, 1, 2, 4, 8, 16 s) during Laser OFF sessions for optogenetic animals (*n* = 8) indicating the rate of discounting (*k *= −0.081). ***D***, Probability of choosing an immediate choice at each delay. A shift away from preference toward the delay lever (0, 1, 2, and 4 s) as delays increase and an equal probability of choosing between immediate and delay levers at the 8 s delay. The horizontal dashed line indicates where animals were equally likely to choose between the immeidate and delay lever.

The sequence of events for a single trial is depicted in Figure 1*A*, for additional detail (Linsenbardt et al., 2017). Briefly, the back wall contained a house light, which signified the start of a trial and remained illuminated for 10 s. Subsequently, levers extend for the initiation, retract (1 s), and re-extend for the choice epoch. Sucrose pellets were then dispensed into the hopper followed by an intertrial interval. Then, 20 mg sucrose pellets were used for electrophysiology and 45 mg sucrose pellets were used for optogenetic experiments.

“Forced trials” were implemented for the immediate and delay levers, where two consecutive responses on the same lever would result in a forced trial for the nonchosen lever on the next trial (Fig. 1*B*). If an animal did not lever press for the forced trial, the forced trial would be repeated until the lever was pressed. There was no effect of forced trials on *i*-value (Fig. 1*B*).

The session terminated either after 30 choice trials or 35 min for the optogenetic experiment (all delays) and for the 0, 1, and 2 s delays in the electrophysiology experiment (in the standard operant chambers). When animals were moved from the standard operant boxes to the custom operant box for the electrophysiological recordings (4 and 8 s delays), sessions terminated after either 40 choice trials or 45 min using 20 mg sucrose pellets to maximize number of trials obtained while recording. The delays were completed in ascending order (0, 1, 2, 4, 8, 16 s) with a day off in between the start of each new delay. Eight to twelve sessions were given at the 0 s delay and four sessions at the 1 and 2 s delay. Nine sessions were completed at the 4, 8, and 16 s delays to account for optogenetic manipulation (Table 1). Inhibition (Laser ON sessions) occurred every other session following the first two sessions in order to acclimate animals to the new delay. Electrophysiological recordings during the 4 and 8 s delay were obtained until a viable signal was no longer apparent. Magnitude discrimination was determined at the 0 s delay in the standard operant chambers using the 45 mg sucrose pellets before animals were accepted for surgery with an exclusion criterion of 80% (4.8 pellets) of the maximum reward value (six pellets) for optogenetic inhibition and 70% criterion for electrophysiological recordings (4.2 pellets). The average value of the immediate lever over the last 10 choice trials was determined for the last 3 d of the 0, 1, and 2 s delay and was used to determine the indifference point of each animal. Animals that were included then either received surgery for optogenetic or electrophysiology experiments (see below, Surgical preparation and implantation, for detail).

**Table 1.** Schedule of optogenetic inhibition for each experimental delay (4, 8, 16 s)

Day 1	Day 2	Day 3	Day 4	Day 5	Day 6	Day 7	Day 8	Day 9
X	X	1	X	2	X	1	X	2
X	X	2	X	1	X	2	X	1

Each X indicates a Laser OFF day. The 1 and 2 indicate Laser ON days (Epoch 1 or 2 inactivation; Fig. 1*A*). Epoch 1 inactivation started once the house light illuminated and terminated at the initiation. Epoch 2 inactivation started at the initiation and terminated at the choice. Epochs 1 and 2 were alternated every other day such that each animal received two “Laser ON days” at both Epoch 1 and 2. See Figure 1 for Epoch 1 and 2 durations prior to choice. Days 1 and 2 were discarded from analyses as animals were adjusting to the new delay and behavior was unstable.

Indifference points for both optogenetic and electrophysiology experiments were obtained by taking the average of the last 10 choice trials of each session for a given delay. For optogenetic inhibition, during delays with optogenetic manipulation (i.e., the 4, 8, and 16 s delays), the last 10 trials of each day for each condition (Laser OFF, Laser ON) were used to determine an indifference point for Laser ON versus OFF conditions at the 4, 8, and 16 s delays. Sessions 1 and 2 were excluded for the Laser OFF condition, as animals were becoming familiar with the new delay and indifferences points were not yet stable. The last 10 trials were taken for each animal for Laser ON and OFF sessions following session two to obtain indifference points. The average of the last 10 trials were used for calculating indifference points for the Laser OFF and Laser ON sessions. The rate of discounting was determined using the Mazur hyperbolic model (Eq. 1; Mazur, 1987):

$$v=\frac{a}{1+kd},$$

here, *v* represents the subjective value of the reward, *a* is the fixed value of the delay reward (6 pellets), *d* is the length of the delay (0, 1, 2, 4, 8, or 16 s), and *k* is the value fit to the hyperbolic function using least squares regression to the indifference points across delays (Fig. 1*C*).

### Surgical preparation and implantation

For all surgeries, animals were placed inside a flow box and anaesthetized with isoflurane gas (2% at 4 L/h) until sedated, at which point they were placed in a stereotaxic frame and maintained on 1–3% isoflurane for the duration of the surgery. Artificial tears were then applied. Subsequently, fur was shaved and the skin at the incision site was sanitized with three rounds of both 70% EtOH and betadine before applying a local anesthetic (Marcaine; 5 mg/kg, s.c.). An anti-inflammatory (Ketofen; 5 mg/kg dose, s.c.) and antibiotic (Cefazolin; 30 mg/kg, s.c.) were injected at the nape of the neck (anti-inflammatory and antibiotic) before beginning the incision. Once the skull was exposed and cleaned of blood, bregma-lambda coordinates were identified. Prior to any implantation (probe or optic fiber), four stainless steel anchoring screws were inserted. Following insertion of either Cambridge Probes or optic fibers, a two-compound dental cement was used to adhere implants to anchoring screws. Following completion of surgical procedures, animals were maintained in a clean heated cage before being returned to the vivarium.

#### Opsin virus delivery and implantation of optic fibers

Two syringe pumps (Pump 11 Elite; Harvard Apparatus) were attached to each arm of the stereotaxic frame and loaded with 2 μl Hamilton syringes (7002KH, Hamilton). Coordinates for ACC viral injections occurred at a 20° angle and were as follows: +3.2 mm AP, +2.0 mm ML, −5.2 mm DV from the bregma. Holes were drilled into the skull to allow the Hamilton syringes to penetrate the brain tissue. Animals then received bilateral injections of 0.65 μl at a flow rate of 0.2 μl/min of the inhibitory Adeno-associated virus (AAV-CaMKIIa-eArchT3.0-EYFP; K. Deisseroth via UNC Vector Core) followed by 10 min of diffusion before retracting the Hamilton syringes. Subsequently, animals received fiber implantation of dual fiber-optic cannulas with guiding sockets (DFC_200/245-0.37_3.3mm_GS1.4_FLT; Doric Lenses).

#### Electrophysiological probe implantation

A rectangular craniotomy was performed over the right hemisphere of ACC (AP: 2.8, ML: 0.3 from the bregma) followed by a durotomy and cleaning/hydration of the probe insertion site with a sterile saline solution. Additionally, two ground screws were placed above the cerebellum. A Cambridge NeuroTech *F* (*n* = 5), *p* (*n* = 4), or *E*-series (*n* = 1) 64-channel silicon probe on a movable drive (Cambridge NeuroTech) was lowered to the target site. Mobility of the movable drive was maintained with a coating of antibiotic ointment.

### Stimulation and recording equipment

#### Optogenetic stimulation

A green (532 nm) laser (MGL-FN-532-300mW; Ultralasers) operated through Med Associates Programming via a TTL (Med Associates) was utilized for stimulation. From the fiber coupler, a mono patch cord (MFP_200/240/900-0.22_1m_FC-FC; Doric Lenses) was attached and traversed the sound-attenuating chambers terminating at the rotary joint (FRJ_1 × 1_FC-FC; Doric Lenses) which attached a Branching Fiberoptic Patchcord (BFP(2)_200/240/ARMO-0.22_0.5m_FCM-GS1.4; Doric Lenses) that was the terminal connection to the animal via guiding socket at the top of the animal's skull. Stimulation did not occur in pulses and remained on for the duration of the epoch to prevent rebound depolarization of cells. Stimulation at the tip of the fiber measured ∼21 mW resulting in predicted irradiance of ∼60 mW/mm^2^ at the fiber tip. Larger irradiance values were opted for in order to traverse the entire region of the ACC with only one fiber per hemisphere.

Optogenetic inhibition (Laser ON) occurred at one of two different epochs during the task for a given session (Epoch 1 inactivation or Epoch 2 inactivation; Table 1 and Fig. 1*A*). Epoch 1 stimulation occurred from the start of a given trial and terminated once an animal initiated the trial (Fig. 1*A*, top). Stimulation remained on if the animal omitted initiating the trial until a response on an initiation lever was made. Epoch 2 stimulation occurred as soon as the animal initiated a trial and terminated once a choice was made (Fig. 1*A*, top). Stimulation remained if the choice was omitted until a choice was made on subsequent trials. Stimulation occurred on the third, fifth, seventh, and ninth session/day of the 4, 8, and 16 s delays to control for carry over effects of the stimulation as well as to obtain indifference points for the Laser OFF condition. All animals received stimulation at both Epoch 1 and Epoch 2 in a cross-over design (Table 1) so that half the animals received Epoch 1 on the third and seventh day and Epoch 2 on the fifth and ninth day and the other half of animals received the opposite configuration. The virus was allowed to express for at least 3 weeks before beginning any optogenetic manipulation.

#### Electrophysiology equipment

Silicon probes were acquired from Cambridge NeuroTech and interfaced with Omnetics connectors (Omnetics). Silicon electrodes were mounted the day prior to surgery to Cambridge NeuroTech microdrives. An Intan RHD SPI cable (Intan) connected the headstage to a Doric Commutator (Doric Lenses) positioned above the operant apparatus. An Open Ephys (Open Ephys) acquisition system was used to collect all electrophysiological data. ANY-Maze (ANY-maze Behavioral tracking software) was used to collect all behavioral and locomotor data. ANY-maze locomotor data was synchronized with Open Ephys via an ANY-maze AMI connected to an Open Ephys ADC I/O board. Med PC behavioral events were also synchronized to the electrophysiological recordings via an Open Ephys ADC I/O board. Following sessions with diminished signal, electrodes were lowered 50 µm.

#### Immunohistochemistry, histology, and anatomical nomenclature

**Figure 2.**
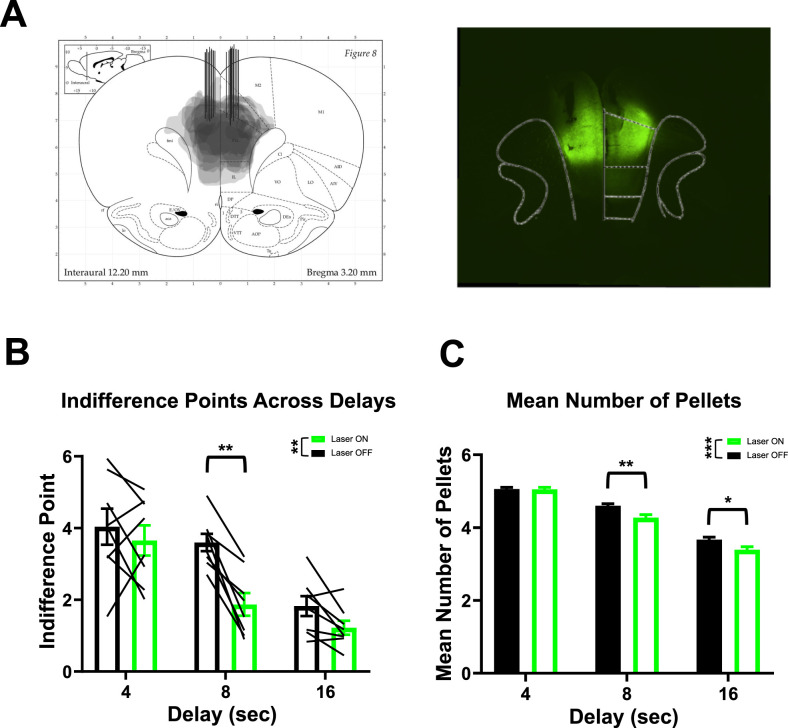
Optogenetic inhibition of ACC prior to choice increases impulsivity measures and is disadvantageous. ***A***, ArchT expression spread and optic fiber placements (left) for all animals (*n* = 8). Representative image of viral spread and optic fiber placements (right). ***B***, Indifference points decrease as delay increases (ANOVA: *F*_(1.48,10.35)_ = 27.53, *p *= 0.0001). Indifference points were decreased on Laser ON (green) sessions compared with Laser OFF (black) sessions (ANOVA: *F*_(1,7)_ = 27.53, *p *= 0.003). Specifically, at the 8 s delay (Holm–Šídák test, *p *= 0.001), indifference points were decreased for Laser ON (green) compared with Laser OFF (black) sessions but not the 4 s (Holm–Šídák tests, *p *= 0.84) or 16 s delays (Holm–Šídák tests, *p *= 0.09). Optogenetic manipulation occurred at the 4, 8, and 16 s delays. ***C***, The average number of pellets earned during choice trials for Laser ON sessions was lower than that during Laser OFF sessions for the 8 and 16 s delays. *p < 0.05, **p < 0.01, ***p < 0.001.

Histological verification of virus and optic fiber implant (Fig. 2*A*) or electrode placements (Fig. 4*A*) was conducted to exclude animals prior to analysis. The locations of each electrode at the end of the experiment are shown (Fig. 4*A*). Most of the recording sites and optogenetic expression ended up in what would be considered rat prelimbic cortex. However, each electrode was mounted on a hyperdrive and lowered when recording quality began to diminish. This corresponded to an average of 0.57 mm (range, 0.125–1.125 mm) of total travel in the D/V axis throughout the experiment. Extrapolating from the ending placement this puts the location of several electrodes in the ACC and possibly M2 according to the 6th Edition of the Paxinos and Watson atlas (Paxinos and Watson, 2007). Therefore, we have adopted the homologous nomenclature of ACC anatomy described in van Heukelum et al. (2020) and refer to the brain region we measure and actuate as ACC.

#### Optogenetics

Animals were perfused within 14 d after behavioral testing with 4% PFA after receiving an anesthetic dose of urethane (1.5–2.0 g/kg). Brains were then fixed in 4% PFA for 24 h before being placed in a 30% sucrose solution (24–72 h) and subsequently stored at −20°C until sliced 50 µm thick. To assess transduction of glutamatergic pyramidal cells within ACC, slices were mounted on gelatin subbed glass slides using an aqueous mounting medium (H-1000-10; Vectashield, Invitrogen). A florescence imaging scope (Nikon Eclipse 80i) was used to verify EYFP-tagged protein expression.

#### Electrophysiology

Animals were anesthetized with urethane (1.5–2.0 g/kg) and subsequently perfused following with 4% PFA after cessation of spinal reflexes. Following tissue extraction, brains were fixed in 4% PFA for 24 h and then transferred to a 30% sucrose solution for cryoprotection. Following our postfix procedures, tissue was stored at −80°C until tissue was sliced at 50 µm and stained for both GFAP and DAPI. Briefly, tissue sections were washed in phosphate-buffered saline (PBS) once. Following this, sections were washed in PBS and 0.1% Triton X-100. Sections were blocked in 1% normal goat serum. Following blocking, the primary antibody (GFAP; goat anti-chicken) was added and allowed to incubate while shaking for 24 h at 4°C. Tissue was washed three times in PBS and then the secondary antibody was added (Alexa Fluor 555; goat anti-chicken). Tissue was incubated and shook in a light-protected box for 2 h at room temperature. Tissue sections were subsequently handled under light-protective materials. Three additional washes in PBS were followed by the addition of DAPI which was allowed to incubate for 10 min at room temperature. Three additional washes in PBS followed. Sections were then mounted on gelatin subbed glass slides with anti-fade mounting medium (sc-516212 Santa Cruz Biotechnology) and imaged to confirm placement across the ACC. Sections were mounted on gelatin subbed glass slides and then imaged to confirm placement across the ACC.

#### Spike sorting

Putative neurons were organized into clusters by Kilosort 2 (Pachitariu et al., 2016). Following automatic spike sorting, supervised curation was performed in Phy2 (https://github.com/cortex-lab/phy). Specifically, it was ensured that the autocorrelograms contained no refractory violations, the waveforms were characteristic of an action potential, and the signal was minimally contaminated by any noise artifacts. Following qualitative characterization in Phy2, data were imported into MATLAB for subsequent analyses. A custom MATLAB routine was used to align spike trains to task events. Spike trains were smoothed using Gaussian convolution with a bin width of 200 ms and *σ* set to 10 ms.

#### Experimental design and statistical analysis

Data were analyzed using custom MATLAB routines in all experiments. To quantify decision-making latency, reaction times from choice latencies were transformed to ranks and graphed as mean ranks. This was done to limit the positive skew of reaction times. All significance *a* values were set at 0.05.

**Figure 3.**
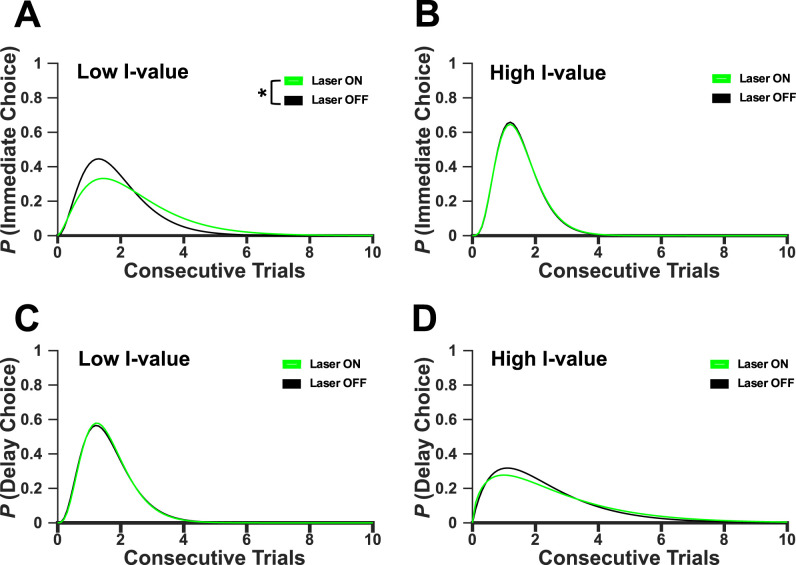
Optogenetic inhibition of ACC increases impulsivity by disrupting ability to deviate from low-value immediate choices at the 8 s delay. Choices were stratified by *i*-value (low, *i*-value <4 and high, *i*-value >3) and choice lever (immediate or delay). Consecutive number of immediate low *i*-value (***A***), immediate high *i*-value (***B***), delay low *i*-value (***C***), and delay high *i*-value (***D***) choices for Laser ON (green) versus Laser OFF (black) conditions were analyzed using a probability density functions (PDF). Optogenetic inhibition increases the consecutive number low *i*-value immediate choices (***A***) at the 8 s delay (Kolmogorov–Smirnov, *p *= 0.022). *p < 0.05.

In the optogenetics experiment, choices were classified into four different types based off choice (immediate or delay) and *i*-value (low, *i*-value <4 and high, *i*-value >3). Distribution of the four choice types were analyzed using a probability density function (PDF) for Laser ON versus OFF conditions (Fig. 3) to determine how consecutive choices are made for immediate and delay choices with high versus low *i*-values. This assessed the ability of the animal to deviate from poor choices, such as choosing the immediate lever when the *i*-value is low or the delay lever with the *i*-value is high.

**Figure 4.**
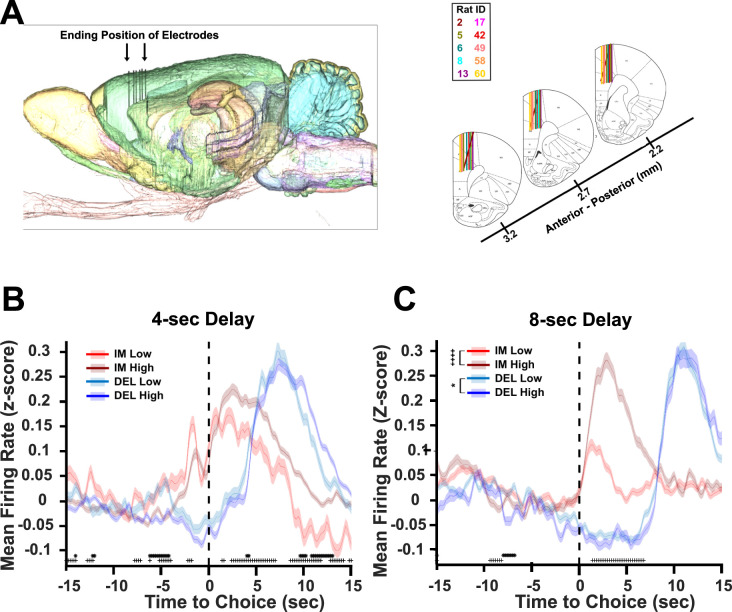
Neural activity of ACC neural populations during immediate and delay choices with low and high *i*-values during the 4 and 8 s delay. ***A***, Electrophysiology placements of silicone probes (***A***, left). Representative image of sagittal slice with probe placement in the right ACC (left) and for all animals (***A***, right). ***B***, ***C***, Grand average mean firing rate for the 4 s (***B***; *n* = 2,120 neurons) and 8 s (***C***) delay (*n* = 2,078 neurons) separated by immediate low and high *i*-value (IM-Low, light blue; IM-High, dark blue) and delay low and high *i*-value (DEL-Low, light red; DEL-High, dark red) choices aligned to the time that the animal presses the choice lever (dashed line at time = 0; low, *i*-value <4 and high, *i*-value >3). Individual timepoints where IM-Low and IM-High firing rates differ (+) or DEL-High and DEL-Low differ (*) as indicated by FDR-corrected *t* tests are marked at the bottom of the graph (***B***, ***C***). Scheffe multiple-comparison tests indicate whether overall firing rates differ between immediate and delay high and low *i*-value conditions within the figure legends (***B***, ***C***). *p < 0.05, DEL-Low versus DEL-High *i*-value; ^++++^*p* < 0.0001, IM-Low versus IM-High *i*-value (***B***, ***C***).

To better understand how firing rates differ for immediate and delay choices with high and low *i*-value, spike trains were aligned to the choice point comprised of an interval of 15 s prior to and 15 s after the choice (−15 to +15 s) and binned at 200 ms, resulting in 151 bins. Using the choice point-aligned spike trains, binned spike counts were smoothed using a moving average filter spanning five bins for each neuron. Firing rates were *z*-scored and the average firing rate for immediate and delay high/low *i*-value trials (low, *i*-value <4 and high, *i*-value >3) were calculated and plotted for the 4 and 8 s delays (Fig. 4*B*,*C*). ANOVAs and post hoc tests were then used to analyze differences between firing rates across trials differing by lever and *i*-value.

**Figure 5.**
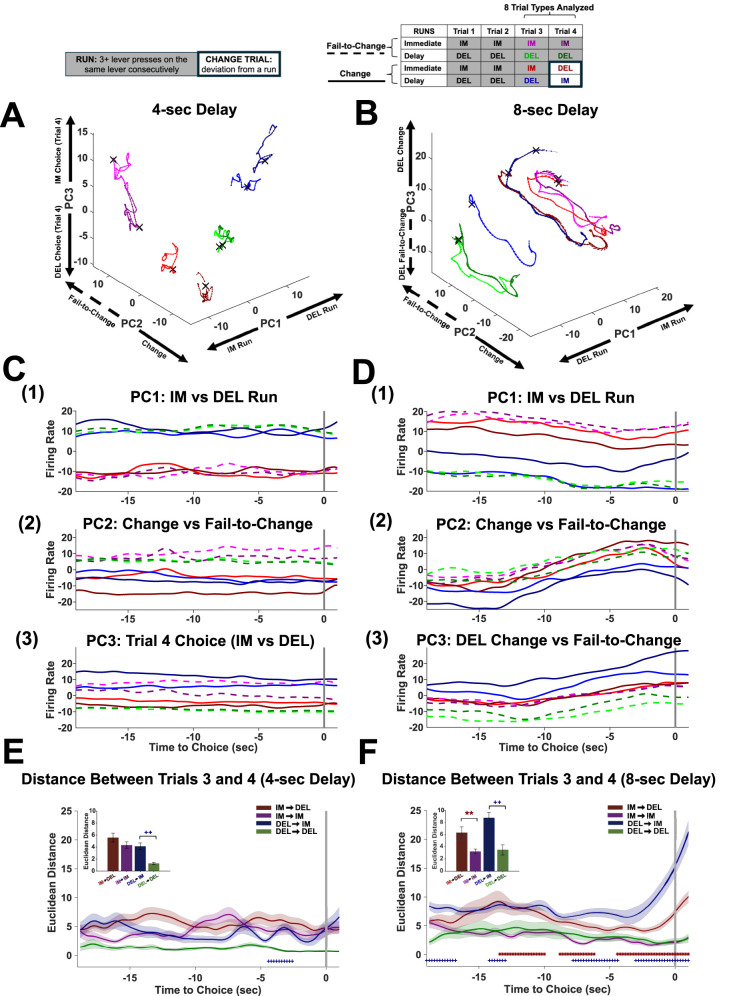
PCA for runs during the 4- and 8 s delays reveal shift from procedural (4 s) to deliberative decision-making (8 s). ***A–F***, Population activity during the third and fourth trial for each run (IM-Change, IM-Fail-to-Change, DEL-Change, DEL-Fail-to-Change, see key) from the 4 s (***A***, ***C***, ***E***) or 8 s (***B***, ***D***, ***F***) delay were analyzed using PCA. Runs consisted of an initial consecutive three choices on either the immediate (IM) or delay (DEL) lever, and the fourth trial consisted of either the “Change” (solid lines; end run) or “Fail-to-Change” (dashed lines; continue run) trial (see key). ***A***, ***B***, Trajectories (101 time bins/points) for the third and fourth trial of the four runs (8 trial types total) were analyzed and plotted in 3D space for each of the top 3 PCs for the 4 s (***A***) and 8 s (***B***) delays. The X denotes the choice point in each trajectory. ***C***, ***D***, Firing rates for each of the eight trial types were plotted for each individual PC for the 4 s (***C*_1–3_**) and 8 s (***D*_1–3_**) reveal that the individual PCs encode different dimensions of the decision-making process such as whether the run was IM or DEL (***C*_1_**, ***D*_1_**) for PC1, run transition (***C*_2_**, ***D*_2_**) in PC2 for the 4 s (***C***) and 8 s delay (***D***); however, the encoding of run transition is less clear at the 8 s delay (***D*_2_**). PC3 encoded trial 4 choice during the 4 s delay (***C*_3_**) and run transitions for DEL runs at the 8 s delay (***D*_3_**). ***E***, ***F***, Change in Euclidean distance (mean ± SEM) between the third and fourth trial across time (***E***, ***F***, choice point indicated by vertical gray line) and average distance (±SEM) between the third and fourth trial trajectories (***E***, ***F***, inset) for each of the four runs during the 4 s (***E***) and 8 s (***F***) delays. Individual timepoints where IM-Change and IM-Fail-to-Change distances differ (red, *) or DEL-Change and DEL-Fail-to-Change (blue, +) as indicated by FDR-corrected *t* tests are marked at the bottom of the graph (***E***, ***F***). ^++^*p* < 0.01, DEL-Change versus DEL-Fail-to-Change; **p < 0.01, IM-Change versus IM-Fail-to-Change.

To better understand the changes in neural activity that underlie how a bias for an option may emerge and be abandoned over the course of a session, consecutive trials were analyzed in the electrophysiology animals. To quantity this, three or more consecutive choices on either lever was considered a “run.” Three consecutive choices were defined as the threshold since the animal continued to choose the same lever despite being exposed to the other lever on a forced trial. Runs were then subdivided by three different criteria. First, runs were split into Change or Fail-to-change runs based on the animal's choice on the fourth trial. When animals chose the opposite lever on the fourth trial, this was referred to as “Change.” When the animal stayed on the same lever for the fourth trial, this was referred to as “Fail-to-Change.” Second, runs were further stratified by the lever the animal chose on the first three trials (i.e., immediate, delay). Third, Trials 3 and 4 were selected for analysis (Fig. 5; see key). Each of the three criteria above resulted in eight different trial types to be analyzed at each delay.

To examine differences in neural activity that contribute to either Change or Fail-to-Change, the third and fourth trial of each type of run was first analyzed using a global principal component analysis (PCA). This PCA allowed us to visualize neural activity patterns that unfold over the entire population of neurons. It was performed separately for the 4 s (Fig. 5*A*,*C*) and 8 s delays (Fig. 5*B*,*D*) and included each dataset within a delay. For all PCAs, spike trains were aligned to the choice point comprising an interval of 19 s prior to and 1 s after the choice (−19 to +1 s) and binned at 200 ms, resulting in 101 bins. Using the choice point-aligned spike trains, binned spike counts were smoothed using a moving average filter spanning five bins for each neuron. Only neurons firing during the third and fourth trial of each type of run were included in the analysis (Table 2; Fig. 5, key) resulting in a time bin X neuron matrix for each of the eight trial types outlined above. Firing rates from each neuron (*n* = 581 4 s, *n* = 1,166 8 s; Table 2) were contained in columns and each of the 101 time bins in rows. The eight matrices were then concatenated and *z*-scored prior to running PCA. For example, the 4 s delay consisted of a matrix (808 × 581) which contained *n* = 581 neurons, where each row corresponded to firing rates at each time bin (101 time bins per trial type, 808 total) and each column corresponded to the firing rate of an individual neuron. PCA was then conducted to analyze neural activity across Trials 3 and 4 of each of the following runs: Immediate-Fail-to-Change, Delay-Fail-to-Change, Immediate-Change, and Delay-Change. The three most explanatory dimensions were chosen [top 3 principal components (PCs); Fig. 5*A–D*].

**Table 2.** Number of neurons and session numbers used in each PCA

Delay	Starting neuron number	Number of excluded neurons	Total neurons used for PCA	Session numbers used for PCA
4 s	2,120	1,539	581	1, 4, 7, 9, 15, 16, 21, 44, 45, 48, 50
8 s	2,078	912	1,166	23, 28, 29, 30, 31, 32, 43, 52, 53, 54, 55, 57, 58

The total neurons, excluded neurons, and end number of neurons are included. Neurons were excluded for not firing in all trial conditions tested in the PCA. See Materials and Methods for exclusion criteria.

A separate PCA was performed to obtain single session trajectories which allowed differences in neural activity between runs to be further quantified and comparisons between the 4- and 8 s delays. PCA was conducted in a similar manner as described above but separate for each session. This PCA was used to obtain neural trajectories for each individual session that allowed analysis of changes in neural activity patterns from the third to fourth trial of each run (Fig. 5*E*,*F*). Each trajectory contained coordinates in PC space corresponding to the top 3 PCs at each time bin (101 × 3). The Euclidean distance was calculated between the third and fourth trial of each run using the trajectory coordinates derived from the top 3 PCs (Fig. 5*E*,*F*). Specifically, for each individual session, the pairwise Euclidean distance between the third and fourth trial for each run was calculated for the top three PCs. The diagonal of the pairwise distances was used as the distance metric. The temporal evolution of change in Euclidean distances prior to the choice (Fig. 5*E*,*F*) for runs were then subjected to statistical analyses via ANOVA and post hoc comparisons.

## Results

### There is an equal probability of choosing the immediate or delay option at the 8 s delay

As the delay increased, animals chose the immediate reward more often (Fig. 1*C*,*D*). Indifference points across all delays (0, 1, 2, 4, 8, 16 s) were assessed in optogenetic animals for the Laser OFF condition to evaluate the effect of delay on reward value without optogenetic manipulation. A Greenhouse–Geisser corrected RM one-way ANOVA indicated a significant effect of delay on indifference points (*F*_(2.19,15.33)_ = 16.64, *p *= 0.0001; Fig. 1*C*). As delays increased, the indifference points decreased, indicating discounting. The Mazur hyperbolic DD curve was fit to indifference points to calculate the rate of discounting (*k *= −0.081).

While initially biased toward the delay lever, the strong preference for the delay lever decreased with increasing delays (Fig. 1*D*), further indicating discounting. The delay impacted the percent of immediate choices made during a session [Kruskal–Wallis (6,120) *χ*^2^ = 79.02; *p *> 0.0001]. The percentage of immediate choices made during a session increased as delays increased. Similar to previous reports from our lab, Wistar rats are equally likely to choose between the immediate and delay level at the 8 s delay (Linsenbardt et al., 2017). These results provide evidence that the preference for the delay lever is no longer observed at the 8 s delay. Collectively, these results indicate the presence of discounting in Wistar rats using this DD paradigm and that more deliberation may be required at the 8 s delay rather than relying on a preference for the delay lever to make decisions.

### Optogenetic inhibition of ACC increases impulsive choices at an 8 s delay

Bilateral expression of ArchT in the ACC was present for all animals included in the analyses with expression throughout the ACC with some ventral spread (Fig. 2*A*). Indifference points for each condition (Laser ON vs Laser OFF) were calculated by averaging the last 10 trials of each session for each delay and a hyperbolic discounting function (Mazur, 1987) was fit to observations in the Laser ON and Laser OFF conditions for the 4, 8, and 16 s delays (curves not shown, Laser ON). To assess differences between Laser ON/OFF conditions an Extra-sum-of-squares *F*-test was used to determine whether one model accurately describes both conditions. One curve did not adequately fit both conditions (Extra-sum-of-squares *F*-test: *F*_(1,46)_ = 10.46, *p *= 0.002), indicating differences in *k*-values between conditions. Lower AUC for the Laser ON than OFF was observed (paired samples *t* test, *t*_(7)_ = 5.3, *p *= 0.001). Collectively these data indicate that inhibition of the ACC increases impulsive responding.

To further assess what drove the differences between the Laser ON/OFF conditions, indifference points were compared at 4, 8, and 16 s delays individually (Fig. 2*B*). Greenhouse–Geisser corrected two-way repeated-measures ANOVA revealed a main effect of delay (*F*_(1.48,10.35)_ = 27.53, *p *= 0.0001) and main effect of Laser ON/OFF condition (*F*_(1,7)_ = 27.53, *p *= 0.003; Fig. 2*B*). Post hoc comparisons were used to assess differences in impulsivity at each delay. No differences were observed between Laser ON versus Laser OFF at the 4 s (Holm–Šídák tests, *p *= 0.84) or 16 s (Holm–Šídák tests, *p *= 0.09) delays (Fig. 2*B*). At the 8 s delay, the indifference points for the Laser OFF condition was larger than Laser ON condition (Holm–Šídák test, *p *= 0.001; Fig. 2*B*). These results indicate that the indifference points between Laser ON/OFF conditions during the 8 s delay were the major factor in differences between impulsivity measures. The selective effect at the 8 s delay may be attributable to the equal probability of choosing either the delay or immediate lever (Fig. 1*D*).

The number of sucrose pellets earned during Laser ON and OFF sessions were assessed across the 4, 8, and 16 s delays to evaluate if increased impulsivity led to fewer rewards. The number of pellets earned during choice trials for Laser OFF and Laser ON sessions differed when collapsed across 4, 8, and 16 s delays (Wilcoxon rank sum tests, *Z *= −3.39, *p* = 0.0007; Fig. 2*C*), with Laser OFF sessions earning more pellets. When stratified by delay, the number of pellets earned for Laser ON and OFF sessions during choice trials differed for both 8 s (Bonferroni-corrected Wilcoxon rank sum tests, *Z *= −2.84, *p* = 0.01; Fig. 2*C*) and 16 s delays (Bonferroni-corrected Wilcoxon rank sum tests, *Z *= −2.55, *p *= 0.03; Fig. 2*C*) but not for the 4 s delay (Bonferroni-corrected Wilcoxon rank sum tests, *Z *= −0.73, *p *= 1; Fig. 2*C*). The number of pellets earned during the 8 and 16 s delays were greater for the Laser OFF sessions, indicating that the increase in impulsive choice driven by optogenetic inhibition of the ACC was disadvantageous.

To further assess whether forced trials impacted number of pellets earned and therefore whether the increase in impulsive choices was disadvantageous, number of pellets on both forced and choice trials were assessed for Laser ON and OFF sessions. When accounting for both choice and forced trials, the number of pellets earned on Laser ON and OFF sessions differed across the 4, 8, and 16 s delays (data not shown; Wilcoxon rank sum tests, *Z *= −1.98, *p *= 0.048). More pellets were earned during the Laser OFF session. Collectively, these results indicate that optogenetic inhibition of ACC increases impulsive choices, resulting in suboptimal decision-making.

We tested our hypothesis that ACC contributes to strategies that impact choice behavior that emerges across trials. To examine choice sequences, trials were split into four different types based on *i*-value (low, *i*-value <4 and high, *i*-value >3) and choice lever (Fig. 3). This assessed the ability of the animal to deviate from poor choices, such as continuing to press the immediate lever when the *i*-value was low. It was hypothesized that optogenetic inhibition would disrupt the ability to switch away from continuing to choose the immediate lever when *i*-value was low or the delay lever when *i*-value was high. For both the Laser ON/OFF conditions the distribution of consecutive choices was determined and a gamma distribution was fit to the data. The Laser ON and Laser OFF conditions did not differ in number of consecutive immediate choices when *i*-value was high (Wilcoxon rank sum tests, *Z *= −0.05, *p *= 0.96; Fig. 3*B*), delay choices when *i*-value was low (Wilcoxon rank sum tests, *Z *= −0.15, *p *= 0.88; Fig. 3*C*), or delay choices when *i*-value was high (Wilcoxon rank sum tests, *Z *= −0.60, *p *= 0.55; Fig. 3*D*). Laser ON conditions lengthened consecutive immediate choices when *i*-value was low during the 8 s delay (two-sample Kolmogorov–Smirnov, *p *< 0.0001, *k* = 0.29; Wilcoxon rank sum tests, *Z *= −2.25, *p *= 0.02; Fig. 3*A*), providing evidence that optogenetic inhibition disrupted ACC signals required to shift away from poor decisions. Collectively these results suggest that, at the 8 s delay, optogenetic inhibition of ACC increases impulsivity by impacting the animals’ ability to update decision-making, specifically deviating from choosing the immediate lever when the value is low.

### Changes in mean firing rate do not explain effects of optogenetics on behavior

To determine why optogenetic inhibition of ACC increased impulsivity at the 8 s delay, male Wistar rats were unilaterally implanted with 64-channel silicone probes in ACC (see Materials and Methods; Fig. 4*A*), and recordings during the 4 and 8 s delay were analyzed. For both the 4 s (Fig. 4*B*) and 8 s (Fig. 4*C*) delays, a Greenhouse–Geisser corrected tree-way repeated-measures ANOVA was performed to evaluate the effect of time (151 bins), lever (immediate or delay), and *i*-value (high or low) on neural firing rates. There was a significant effect of time (*F*_(4.61,6,102)_ = 125.09, *p *< 0.0001) but not *i*-value (*F*_(0.031,40.68)_ = 0.81, *p *= 0.37) or lever (*F*_(0.031,40.68)_ = 0.53, *p *= 0.47) on firing rates at the 4 s delay (Fig. 4*B*). There were significant effects of time (*F*_(3.50,4,344.13)_ = 74.24, *p *< 0.0001) and lever (*F*_(0.023,28.96)_ = 11.37, *p *= 0.0008) but not *i*-value (*F*_(0.023,28.96)_ = 1.49, *p *= 0.22) on firing rates at the 8 s delay (Fig. 4*C*). However, at both the 4 and 8 s delays, significant interactions between time, lever, and *i*-value (4 s, *F*_(4.61,6,102)_ = 9.18, *p *< 0.0001; 8 s, *F*_(3.50,4,344.13)_ = 17.80, *p *< 0.0001) were detected. Firing rates varied depending on time, *i*-value, and choice levers for both 4 and 8 s delays.

Peaks in firing rates for immediate choices were shifted to earlier timepoints than delay choices, and higher firing rates were observed for immediate high *i*-value compared with low *i*-value choices following the choice point. Benjamini–Yekutieli false discovery rate (FDR)-corrected *t* tests at each timepoint were conducted to determine the timepoints at which firing rates for high versus low *i*-value differed for the immediate and delay lever. Differences between firing rates for high and low *i*-value choices on a given lever were more consistent during the 4 s (Fig. 4*B*) than the 8 s (Fig. 4*C*) delay. Notably, during the 8 s delay, differences in firing rates for immediate choices stratified by *i*-value were greatest following the choice point (Fig. 4*C*). The infrequency of differences prior to the choice at the 8 s delay was not consistent with the effects of optogenetics at this delay and therefore we analyzed ensemble activity in these recordings.

### ACC networks shift from procedural encoding at 4 s to deliberative encoding at 8 s delay

Runs were defined as three or more consecutive choices on the same lever followed by either continuing the run on the fourth trial (Fail-to-Change run) or changing levers and ending the run on the fourth trial (Change run) for the immediate and delay levers (see Materials and Methods). To assess neural activity associated with decision-making, ensemble analyses were conducted on spike trains 19 s prior to and 1 s after the choice was made on a given trial type (Fig. 1*A*, bottom). Additionally, to better understand neural activity related to shifts in choices, the remainder of the analyses focused on Change or Fail-to-Change runs (see Materials and Methods; Figs. 1*B*, 5, key). Neural activity was assessed between the third and fourth choice trial in the run (Fig. 5) where the fourth choice could either be to continue to the run (Fail-to-Change) or to shift to the opposite lever (Change). PCA was performed across each trial type on neurons for the 4- and 8 s delays to obtain the neural trajectories of the third and fourth trial of runs. This enabled an assessment of whether neural signatures of continuing with the current run (IM/DEL Fail-Change) differed from those of choosing to abandon the current run on the fourth trial (IM/DEL Change).

### PCA reveals qualitatively different trajectories in state space across 4 and 8 s delays

The top three PCs and the trajectories for the eight trial types corresponding to the third and fourth trial of each run are plotted for the 4 s (Fig. 5*A*,*C*) and the 8 s delays (Fig. 5*B*,*D*). The top 3 PCs explained 65% of the variance for both the 4- and 8 s delays. For the 4 s delay, each of the first three PCs clearly separated features of the task (Fig. 5*A*,*C*); where PC1 reflected neural activity patterns related to which lever the animal chose on the run (immediate vs delay; Fig. 5*C*_1_), PC2 separated if the animal changed their choice (Change vs Fail-to-Change; Fig. 5*C*_2_), and PC3 separated what choice the animal made on the fourth trial (immediate vs delay lever; Fig. 5*C*_3_). At the 8 s delay, PC1 still reflected the lever the run was taking place on (Fig. 5*D*_1_). However, PC2 less clearly reflected if the animal would change their choice (Fig. 5*D*_2_), and PC3 reflected DEL-change or DEL-Fail-to-Change sequences (Fig. 5*D*_3_). We also observed that each PC did not vary much in time during the 4 s delay (Fig. 5*C*) but exhibited ramping-like activity prior to the choice point during the 8 s delay (Fig. 5*D*). Collectively, these data indicate that the neural dynamics in ACC during decision-making differs across the delays of the DD task. Specifically, at the 4 s delay, the stability of the PCs in time (Fig. 5*C*) and clearly defined task features (Fig. 5*A*,*C*) are consistent with a procedural decision-making strategy where pre-established evidence is used to guide decisions. In contrast, at the 8 s delay, ramping-like activity in the neural trajectories prior to the choice (Fig. 5*D*) is more consistent with evidence accumulation during a deliberative strategy.

### Euclidean distances are larger for change versus fail-to-change runs

To quantify differences in how ACC encodes Change versus Fail-to-Change trials at different delays, Euclidean distances between the trajectories from the third and fourth trials were calculated for each run using PCA from individual sessions (Fig. 5*E*,*F*; see Materials and Methods). The rationale for this approach is that updates to a decision-making strategy should require distinct neural activity patterns on the fourth trial. If the trajectories occupy similar PC space between trials (small Euclidean distance), this reflects little change in the neural activity patterns between the third and fourth trials, while large distances reflect larger changes in neural activity. We therefore hypothesized that larger distances between the third and fourth trial would be observed for Change trials compared with their Fail-to-Change counterparts. We also hypothesized that distance measures for Change runs would differ for the 4- and 8 s delay, based on the results of the optogenetics experiments. To test these hypotheses, we evaluated the change in Euclidean distance between trajectories for each run across time.

A Greenhouse–Geisser corrected three-way repeated-measures omnibus ANOVA was run on Euclidean distance between trajectories in PCA space to evaluate the effects of time (101 bins) and runs (IM and DEL, Change and Fail-to-Change) and delay (4- and 8 s). There was a significant effect of delay (*F*_(0.016,0.35)_ = 23.41, *p *< 0.0001), runs (*F*_(0.047,1.04)_ = 20.59, *p *< 0.0001), and time (*F*_(1.57,34.53)_ = 6.83, *p *< 0.0001) on Euclidean distance measures. There was also a significant three-way interaction between time, runs, and delay (*F*_(4.71,103.59)_ = 4.11, *p *= 0.001) indicating the temporal evolution of the runs differed across delays.

### Euclidean distances differ more on DEL-change runs at the 8 s versus the 4 s delay

This interaction was further interrogated by post hoc comparisons to assess the differences between 4- and 8 s delays runs (Fig. 5*E*,*F*, insets). In support of our hypothesis, larger changes in Euclidean distance of DEL-Change and DEL-Fail-to-Change runs were observed during the 8 s delay when compared with the 4 s delay (Scheffe tests: *p *< 0.0001, DEL-Change; *p *= 0.007, DEL-Fail-to-Change). However, no differences in Euclidean distance were observed between delays for the IM-Change or IM-Fail-to-Change runs (Scheffe tests: *p *= 0.43, IM-Change; *p *= 0.07, IM-Fail-to-Change).

### Euclidean distances are stable at the 4 s delay and dynamic at the 8 s delay

To further investigate our observation that distance metrics are generally larger for Change versus Fail-to-Change runs, we then evaluated whether runs differ within each delay. To assess the effects of time (101 timebins) and runs (IM and DEL, Change and Fail-to-Change) on Euclidean distances, a Greenhouse–Geisser corrected two-way repeated-measures ANOVA was run separately for each delay.

At the 4 s delay, there was a main effect of run (*F*_(0.040,0.40)_ = 10.36, *p *= 0.0003) but no effect of time (*F*_(1.34,13.36)_ = 1.77, *p *= 0.15) on Euclidean distance measures (Fig. 5*E*). However, the interaction between time by run was significant (*F*_(4.00,40.08)_ = 2.90, *p *= 0.03), indicating that although differences were detected between run types, the runs were relatively stable across time with some run types varying more than others. Post hoc comparisons were used to assess the differences between Change and Fail-to-Change runs for the DEL and IM levers (Fig. 5*E*, inset). In support of our hypothesis, the Euclidean distance between the third and fourth trial was larger for DEL-Change than DEL-Fail-to-Change (Scheffe tests: *p *= 0.007) but not for IM-Change versus Fail-to-Change runs (Scheffe tests: *p *= 0.17). The lack of differences between the IM-Change and Fail-to-Change runs at the 4 s delay may partially explain the effects seen during the optogenetic experiment and further support our hypothesis that at the 4 s delay, decisions are more procedural and guided by the delay lever preference.

At the 8 s delay, there was a main effect of run (*F*_(0.030,0.36)_ = 18.78, *p *< 0.0001) and time (*F*_(1,12.04)_ = 10.28, *p *< 0.0001) on Euclidean distance measures as well as a significant time by run was interaction (*F*_(3.01,36.13)_ = 7.43, *p *< 0.0001; Fig. 5*F*). These results suggest that the 8 s delay is more dynamic than the 4 s delay, which is consistent with the temporal evolution of the PCs (Fig. 5*C*,*D*). Further supporting our hypothesis that larger distances would be observed for Change compared with Fail-to-Change runs, post hoc comparisons indicated that the Euclidean distance between the third and fouth trial was larger for both DEL (Scheffe tests: *p *= 0.002) and IM (Scheffe tests: *p *= 0.004) Change compared with their respective Fail-to-Change counterparts (Fig. 5*F*, inset). These data suggest that at the 8 s delay, updating is required for both IM and DEL-Change runs (Fig. 5*F*). Notably, these data contrast the results observed at the 4 s delay (Fig. 5*E*), where large changes in neural activity patterns are only evident when switching away from the preferred lever.

### Euclidean distances increase prior to choice on 8 s delay change runs

Benjamini–Yekutieli FDR-corrected *t* tests at each timepoint were conducted to determine the timepoints at which Euclidean distance differed between IM/DEL Change and Fail-to-Change runs during each delay. DEL-Change differed from DEL-Fail-to-Change more consistently during the 8 s delay (Fig. 5*F*) than the 4 s delay (Fig. 5*E*) and was especially pronounced leading up to the choice. IM-Change also consistently differed from IM-Fail-to-Change at the 8 s delay (Fig. 5*F*). These data further support our hypothesis that encoding of runs differ by delay and provides an explanation for why optogenetic inhibition had effects at the 8 s, but not 4 s, delay.

### Reaction times at 8 s are consistent with deliberative decision-making

**Figure 6.**
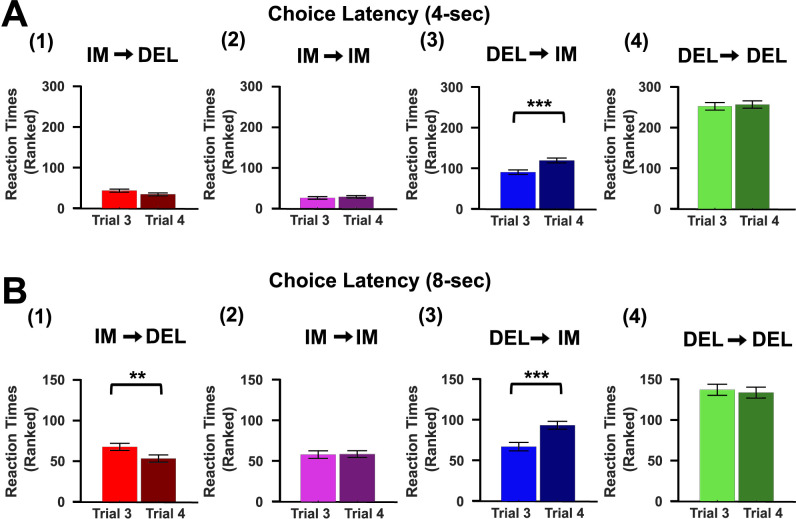
Choice latencies between the third and fourth trial of runs provide evidence of a shift from procedural (4 s) to deliberative decision-making (8 s). ***A***, ***B***, Choice latencies (mean rank ± SEM) compared for third and fourth trial for each of the four runs at the 4 s (***A***) and 8 s (***B***) delays. ***A***, Choice latencies increase on the 4th compared with 3rd trial during the DEL-Change run (***A*_3_**, Wilcoxon signed-rank test: *Z *= −3.74, *p *= 0.0002). No differences in choice latencies were observed between the third and fourth trial for any other run (***A*_1_**, ***A*_2_**, ***A*_4_**, Wilcoxon signed-rank test: *Z*'s < 1.72, *p*'s > 0.09). ***B***, Choice latencies significantly differed between the third and fourth trial for the IM-Change (***B*_1_**, Wilcoxon signed-rank test: *Z *= 2.74, *p *= 0.006) and DEL-Change (***B*_3_**, Wilcoxon signed-rank test: *Z *= −3.82, *p *= 0.0001) runs. No differences in choice latencies were observed between the third and fourth trial for the Fail-to-Change runs (***B*_2_**, ***B*_4_**, Wilcoxon signed-rank test: *Z*'s < 0.12, *p*'s > 0.90). **p < 0.01, ***p < 0.001.

If deliberation was more prevalent at the 8 s than 4 s delay, then changes in response latencies should be observed between the third and fourth trials of runs. At the 4 s delay, animals increased choice latencies between the third and fourth trial for DEL-Change runs (Wilcoxon signed-rank test: *Z *= −3.74, *p *= 0.0002; Fig. 6*A*_3_) but not for Fail-to-Change (Wilcoxon signed-rank tests: DEL-Fail-to-Change, *Z *= −0.38, *p *= 0.70, Fig. 6*A*_4_; IM-Fail-to-Change, *Z* = −0.87, *p *= 0.38, Fig. 6*A*_2_) or IM-Change runs (Wilcoxon signed-rank tests: *Z *= 1.72, *p *= 0.09; Fig. 6*A*_1_). This indicates that latencies increase only when switching away from the preferred (delay; Fig. 1*D*) lever at this delay (Fig. 6*A*). In contrast, at the 8 s delay, choice latencies differed between the third and fourth trial for both the IM-Change (Wilcoxon signed-rank tests: *Z *= 2.74, *p *= 0.006; Fig. 6*B*_1_) and the DEL-Change (Wilcoxon signed-rank tests: *Z *= −3.83, *p *= 0.0001; Fig. 6*B*_3_) runs but not for either the IM (Wilcoxon signed-rank tests: *Z *= 0.09, *p *= 0.93; Fig. 6*B*_2_) or DEL (Wilcoxon signed-rank tests: *Z *= 0.12, *p *= 0.90; Fig. 6*B*_4_) Fail-to-Change runs (Fig. 6*B*). While the probability of choosing an immediate or delay choice is roughly equivalent at the 8 s delay (Fig. 1*D*), animals may still retain biases toward the delay lever that aide in deliberating between the two choice options. Responses were faster when going from IM→DEL and slower when going from DEL→IM and therefore consistent with the animals either switching to or away from their previously preferred option (delay lever) prior to the 8 s delay, which is consistent with deliberating an easy or difficult choice, respectively.

## Discussion

The main finding of this study is that two different decision-making strategies are observed in this task; when a clear preference for the delay lever exists (4 s delay), ensembles in ACC reflect procedural decision-making that changes to a deliberative decision-making strategy when a choice preference no longer exists (8 s delay) and is reflected by differences in neural activity associated with runs (Fig. 5). This was supported by changes in reaction times when animals update decision-making strategies (Fig. 6). Optogenetic inhibition of ACC increased impulsive choices exclusively at the 8 s delay; more specifically, it increased the number of consecutive low-value choices. This suggests that disrupting ensemble activity required for deliberative encoding increases measures of impulsivity by preventing updates required to change runs. Collectively, these data indicate that ensemble activity in ACC shifts based on the cognitive demands of the task and impulsive choices emerge from failure to engage a deliberative decision-making strategy.

One explanation for animals failing to update behavior is that optogenetic inhibition of ACC disrupted deliberative processes that were more prevalent during the 8 s delay. Decisions between the immediate and delay choices are most difficult at the 8 s delay given that the number of immediate and delay choices are roughly equal (Linsenbardt et al., 2017; Fig. 1*D*). A number of studies have indicated that ACC is critically involved in difficult decisions requiring more deliberation and less deliberation occurs for difficult decisions when the ACC is inhibited (Papale et al., 2012; G. J. Peters et al., 2013; Peters and Smith, 2020; Schmidt et al., 2019).

The differences in ACC ensemble activity surrounding runs between delays were uncovered by examining PC spaces. When a clear preference for the delay lever exists (4 s delay; Fig. 1*D*), neural activity across PC spaces were relatively static leading up to the choice (Fig. 5*C*,*E*). This indicates that ACC neurons distribute the encoding of task features across the population of neurons in a static manner, which existed prior to optogenetic inactivation (10 s prior to the choice). This may explain the lack of effect of the optogenetic inhibition at the 4 s delay—any information that ACC might contribute to the decision at this delay preceded the inactivation. In addition, a procedural strategy provides an efficient way to arrive at a good decision by limiting the need for deliberation and using evidence that was previously established.

In contrast, at the 8 s delay, neural ensembles were more dynamic prior to the choice on Change runs (Fig. 5*D*,*F*), which also corresponded to the period of time that ACC was inhibited. These data are consistent with the view that, during DD, ACC plays a critical role in decision-making strategies (Powell and Redish, 2016). Our data support and extend this view by indicating that the ACC may be uniquely involved in decisions requiring deliberation.

The behavior of the trajectories in the PC spaces provides insight into how ACC might implement the computations that control decision-making at each delay. While the differences across each delay were robust, a limit to making inferences about these spaces is the shortcomings of PCA. While useful for dimensionality reduction, PCA may not be sufficient to capture dynamics that occur in a high dimensional space given the linear nature of the algorithm (Whiteway and Butts, 2019). Nonetheless, several important inferences about computation have been made using PCA that are supported by analysis tools better equipped to describes dynamics in a high-dimensional space (Durstewitz, 2017).

The differences in the PC spaces across delays suggest that each delay exhibits a different degree of stability. The neural trajectories for each of the trial types at the 4 s delay were restricted to a discrete region of state space (Fig. 5*A*) that were generally well separated from other trial types, suggesting the existence of several meta-stable states—collectively referred to as “multistability” (Brinkman et al., 2022). This suggests that multistability may be necessary for procedural decision-making. In line with this view, prior work from our group examined ACC neural dynamics in well-trained animals during a foraging-based decision-making task (Lapish et al., 2008; De Falco et al., 2019). These studies found that neural trajectories in ACC track task variables by moving through several meta-stable states that reflect discrete features of the task. This is reminiscent of the behavior of the neural trajectories in the current study at the 4 s delay where procedural encoding was observed.

Procedural encoding initially seemed to be present at the 8 s delay. However, at ∼12 s prior to the choice, PC spaces begin to evolve where PC2 and PC3 each begin to move upward (Fig. 5*D*_2_,*D*_3_). The change in the way neural trajectories move through state space at this time suggests a change in the dynamic properties of ACC networks. We speculate that this change reflects a bifurcation in the systems dynamics that is required to perform deliberation. After this time, neural trajectories seem to take linear paths that give way to rotational paths near the choice. This type of linear to rotational dynamics is reminiscent of that observed in prefrontal networks of the nonhuman primate, which has been suggested to correspond to decision commitment (Mante et al., 2013; Aoi et al., 2020).

Collectively, these observations form the basis of hypotheses that can be directly tested with modern techniques to reconstruct latent dynamics from neural recordings (Durstewitz et al., 2010; Balaguer-Ballester et al., 2011a,b; Durstewitz, 2017). Specifically, if a bifurcation exists and options are encoded via attractor dynamics, characterizing this will provide mechanistic insight into how deliberation is implemented in ACC networks. This would also be important to understand how breakdowns in the computations responsible for deliberation result in impaired decision-making. Identifying methods to repair these computations could be a powerful approach to reduce impulsivity and thereby improve treatment outcomes in several psychiatric disorders.

## Synthesis

Reviewing Editor: Mark Laubach, American University Decisions are customarily a result of the Reviewing Editor and the peer reviewers coming together and discussing their recommendations until a consensus is reached. When revisions are invited, a fact-based synthesis statement explaining their decision and outlining what is needed to prepare a revision will be listed below. The following reviewer(s) agreed to reveal their identity: Nandakumar Narayanan. Note: If this manuscript was transferred from JNeurosci and a decision was made to accept the manuscript without peer review, a brief statement to this effect will instead be what is listed below.

Thank you for transferring your study to eNeuro!

Based on the reviews at JN and your revisions, I requested a quick review of your manuscript by an expert on the rodent prefrontal cortex. The reviewer agreed with my assessment. They wrote that your study is "well-done and well-written" and your "interpretations make sense given the data".

I asked the reviewer about two specific aspects of your paper.

First, you chose to frame the study as done in the ACC. But what you show in the paper is PLC (prelimbic cortex). This issue was raised by reviewers at JN.

The reviewer wrote: "The work is focused on PL, and the authors refer to it as ACC. I would acknowledge the anatomy in the discussion."

We would like to ask you to reconsider using the term "ACC" to describe the study. If you have a specific reason for using this term, please explain the rationale in your response to this review and also add a brief explanation to the paper.

Second, it was not clear to me on my initial reading of your paper what you meant by "procedural vs. deliberative strategies".

The reviewer agreed that the terms were not clear. They wrote:

"I think I understand the evidence for the procedural vs. deliberative strategies, but explicit definitions of what the authors mean might help. I would suggest adding these definitions to intro and discuss how their task engages procedural vs. deliberative (i.e., reflecting on prior choices to guide behavior)."

Finally, I was not able to find any links to data or analysis code used in the study. As you indicated financial support from the NIH, would you be able to post your data and code to a public repository? An example of how you did the behavioral analysis in the first part of the study and how you used PCA later in the paper might be good to share.

Please make minor revisions to address these points, and I will review your revised paper.

## Author Response

eNeuro response to editorial review We thank the editor for the helpful comments. We think that this has added clarity and greatly benefitted our manuscript. Comments from the editor are in black text. Author responses are in blue text. In addition, we have highlighted the most recent changes in bold text to the resubmitted manuscript.

Based on the reviews at JN and your revisions, I requested a quick review of your manuscript by an expert on the rodent prefrontal cortex. The reviewer agreed with my assessment. They wrote that your study is "well-done and well-written" and your "interpretations make sense given the data".

I asked the reviewer about two specific aspects of your paper.

First, you chose to frame the study as done in the ACC. But what you show in the paper is PLC (prelimbic cortex). This issue was raised by reviewers at JN.

The reviewer wrote: "The work is focused on PL, and the authors refer to it as ACC. I would acknowledge the anatomy in the discussion." We would like to ask you to reconsider using the term "ACC" to describe the study. If you have a specific reason for using this term, please explain the rationale in your response to this review and also add a brief explanation to the paper.

We appreciate the suggestion. Here is the rationale for using the term ACC. The figure in the manuscript showing the electrophysiology placements represents the location of the electrodes at the end of the experiment. Each electrode was mounted on a hyperdrive and lowered when recording quality began to diminish. This corresponded to an average of 0.57 (range=0.125-1.125) millimeters of total travel in the D/V axis throughout the experiment. Extrapolating from the ending placement this puts the location of several electrodes in the ACC and possibly even M2 according to prior versions of Paxinos and Watson atlas, which still use the term prelimbic. Therefore, we view the term prelimbic as inaccurate. The homologous definition of ACC provided by van Heukelum et al. (2020) accommodates the small, but potentially meaningful, diversity in our recording and optogenetics locations and therefore we have chosen to use this term. We are open to further discussion on this issue if needed.

We have updated the manuscript to better articulate our rationale. We have added the following to the Methods section in the subsection entitled Immunohistochemistry, Histology, and Anatomical Nomenclature Lines 253-264 in the Methods: "Most of the recording sites and optogenetic expression ended up in what would be considered rat prelimbic cortex. However, each electrode was mounted on a hyperdrive and lowered when recording quality began to diminish. This corresponded to an average of 0.57 (range=0.125-1.125) millimeters of total travel in the D/V axis throughout the experiment. Extrapolating from the ending placement this puts the location of several electrodes in the ACC and possibly M2 according to the 6th Edition of the Paxinos and Watson atlas (Paxinos and Watson 1998). Therefore, we have adopted the homologous nomenclature of ACC anatomy described in (van Heukelum et al. 2020) and refer to the brain region we measure and actuate as ACC." Paxinos G, Watson C. The Rat Brain. San Diego, CA: Academic Press, Ltd; 1998 van Heukelum S, Mars RB, Guthrie M, Buitelaar JK, Beckmann CF, Tiesinga PH, Vogt BA, Glennon JC, Havenith MN. Where is cingulate cortex? A cross-species view. Trends in Neurosciences. 2020 May 1;43(5):285-99.

Second, it was not clear to me on my initial reading of your paper what you meant by "procedural vs. deliberative strategies".

The reviewer agreed that the terms were not clear. They wrote: "I think I understand the evidence for the procedural vs. deliberative strategies, but explicit definitions of what the authors mean might help. I would suggest adding these definitions to intro and discuss how their task engages procedural vs. deliberative (i.e., reflecting on prior choices to guide behavior)." We have added the following definition at the end of the second paragraph of the Intro. Both the second and third paragraphs are dedicated to describing how deliberative and procedural decision-making may influence DD behavior.

Lines 54-75 in the Introduction: "In sum, we define deliberative decision-making here as an effortful process to compare the value of available options, whereas procedural decision-making employs rules or strategies that limit the need for deliberation." Finally, I was not able to find any links to data or analysis code used in the study. As you indicated financial support from the NIH, would you be able to post your data and code to a public repository? An example of how you did the behavioral analysis in the first part of the study and how you used PCA later in the paper might be good to share.

Absolutely. We plan to make all data and code publicly available on Figshare upon final publication.

Please make minor revisions to address these points, and I will review your revised paper.

Journal of Neuroscience response to review We thank both reviewers for their helpful and insightful comments. We have made several updates to the manuscript based on their suggestions. In particular, we have provided an extensive rewrite of the manuscript to streamline it and enhance clarity. We feel that the manuscript is much improved and hope the reviewers will agree.

The requested document highlighting changes to the text can be found in the document labeled: "RevisionsHighlighted_Impulsive_choices_emerge_when_anterior_cingulate_cortex_fails_to_encode_deliberative_strategies" • Given the extensive rewrite of the manuscript, we have made a point to comment on where large portions of the text were changed and how reviewer comments were addressed within these sections. In addition, we have highlighted portions of the text in blue font where we have done extensive rewrites. • A clean version of the document can be found under the title: "Impulsive_choices_emerge_when_anterior_cingulate_cortex_fails_to_encode_deliberative_strategies" Response to Reviewers: The reviewer comments are in black text and our responses are in blue text.

Manuscript Instructions: "During discussion of the manuscript, it was noted that the dose of isoflurane anesthesia reported is lower than is typically used. Please check the isoflurane percentage reported for accuracy, and if the current values are accurate, please provide relevant details on how the level of anesthesia was assessed during surgery." Thank you for calling our attention to this oversight this has been fixed to read 1-3% (see line 180) "On p. 22, Supplemental Materials and Methods are cited. It appears that there is no supplemental materials section, which conforms with the Journal's guidelines, so the text should be corrected. " Thank you for catching this. We have removed all references to supplemental materials Reviewer Comments:

Reviewer #1 Rationale for Significance Rating for Authors: "the experiments are interesting and present evidence corroborating that coordinated medial prefrontal cortex activity is necessary for the establishment of cognitive stability, and that disrupting this activity promotes cognitive flexibility (impulsivity, in this task design). However, the complexity of what the authors are attempting to convey in their analyses is hampered by a lack of clarity in the writing at the conceptual level, both in the introduction and discussion motivating the experiments, and in the results presenting the findings." We thank the reviewer for their positive view of the study. As described below, we have provided a complete reworking of the manuscript with a focus on clarifying and simplifying the results while retaining rigor.

Comments to the authors:

R1.1: "This manuscript by White and colleagues investigated the role of dorsomedial prefrontal cortex (dmPFC) in regulating decisions in delay discounting, in particular comparing when they are less stable versus more stable. Using both optogenetic methods and single unit recordings, they say that they have found that uncertain choice preferences triggered by a long delay are associated with unstable firing patterns, and are susceptible to optogenetic disruption to provoke selection of a less valuable, less delayed reward. In contrast, when choices were stable for selecting a shorter delay, presumably requiring less deliberation, ensemble activity also appeared more stable and optogenetic silencing was ineffective. The overall impression I have of this manuscript is that the experiments are interesting and present evidence corroborating that coordinated medial prefrontal cortex activity is necessary for the establishment of cognitive stability, and that disrupting this activity promotes cognitive flexibility (impulsivity, in this task design).

Thank you for the enthusiasm.

R1.2: However, the complexity of what the authors are attempting to convey in their analyses is hampered by a lack of clarity in the writing at the conceptual level, both in the introduction motivating the experiments (see next paragraph), and in the results. I just found it surprisingly hard to read, both in the text and in the figures, and this impeded my ability to evaluate the claims the authors were making.

We are in complete agreement and have taken several major steps to improve the clarity of the manuscript (see below).

R1.3: Although the sentences are well constructed, the paragraphs across all phases - introduction, results, discussion - do not seem to connect well to one another. For example, there are of course a wide variety of cognitive constructs that could be used to describe these data, and several of them are used in the abstract, more in the introduction, and more in the results, including: impulsivity, prospection, deliberation, procedural/rule based responding, planning, schema encoding etc. These concepts are all relevant and interrelated, and some have been put in conflict with each other, but these interrelationships and conflicts aren't presented in a way that lets us know whether this paper provides data to clarify the issue. Likewise, the discussion of prior dmPFC data presents the idea that some papers have found effects, but others not, without giving us context as to why the authors think this might be the case. Overall, the manuscript gives the impression that the authors are uncertain about how to discuss and present their data. This made it slightly difficult to tell when the paper was getting to the point, or what hypothesis a given analysis was trying to address in the results, or what the goal of these experiments was in the discussion, and impeded my progress through the manuscript.

We have conceptually focused the manuscript on the aspects of decision-making that are closest to the data. We have removed concepts like prospection and planning, as we felt these were obfuscating our message. We think that these concepts are important, but require more experiments to better ground them in data. We think that refining and cutting back on the concepts defined to only include the necessary terminology improved the flow of the paper and the ability to interpret results.

The complete rewrite now clearly addresses the goals of the experiment and our underlying hypotheses. Furthermore, your observation about the connectivity of paragraphs across all phases is well-taken. We've streamlined our discussion to include only necessary terminology directly supported by our data. We've also ensured clear articulation of our hypotheses, linking each analysis explicitly to our research goals. • We have addressed the oversight in the discussion of mixed effect in DD when investigating dmPFC (see lines 74-78) to now include our interpretation of why mix effects have been observed.

Overall, these revisions have significantly improved the manuscript's flow and interpretation ease. We appreciate your guidance in refining our work.

R1.4: The behavioral results swing between being rather jargony ("planning index", and to some extent "i value" and extremely literal (comparison of 8s and 4 second conditions). Sometimes the paper could help us interpret its findings more in the results section, to motivate the next analysis that will be employed. For example, I appreciate the detailed analysis of behavior that the Planning Index in Figure 2 reflects. However, I can't advocate for using the Planning Index itself, at least not without a great deal more explanation. It is a fairly opaque measure that obscures what is actually being measured (how much the animals respond on the same lever twice in a row for each trial type) and replaces it with a psychological concept of "planning" and a normalized measure that is not commonly used (to my knowledge) or self-evident in its meaning when looking at the figure itself. I do think that a measure that describes what is actually happening could be revealing, particularly given that the tendency for animals to stick to one lever or switch to another is highly relevant to the structure of their electrophysiological analyses. It would also be helpful for connecting to literature on the relevant circuitry for controlling what side an action occurs on, etc. I'd recommend still showing these data, but less "normalized" and more explained - including deciding whether to connect this behavior to the other concepts in the introduction.

Thank you for the suggestion. After careful consideration, we have elected to remove all the analysis of the planning index and the discussion of prospection that accompanies it. The inclusion of this concept was an extension of our prior work and guided our hypotheses when performing these studies. However, these hypotheses were not supported which led to a somewhat awkward transition in the paper between the behavior, optogenetic, and electrophysiology data. To address this, we removed this analysis and provided additional behavioral analysis necessary to understand the electrophysiology data. We feel that this adds clarity, and we hope the reviewer will concur.

R1.5: The lack of clarity becomes more challenging when the manuscript moves into analysis of its electrophysiological findings. The results section wants the reader to compare PC structure, trajectories, and choice latencies across 4 second and 8 second trials, but the figures and statistics are not at all arranged in a way that makes it straightforward to do so, by having all the 4 second data in one figure and all the 8 second data in the other. The figures are hard to read in other ways- for example, in PCA space in part of Figure 6A and 7A (which each have multiple panels) the choice point is marked with an X, as noted in the figure caption, but the figures themselves also have large arrows (probably because the X are hard to see) which were not indicated in the caption. Other aspects of the figures make it somewhat difficult to keep track of the findings, eg the need to refer back to which combination of colors and dashed lines were which kind of trial was very taxing, especially when this was coupled with the repeated desire of the authors to compare these lines in Figure 6 with lines in Figure 7.

We have completely reworked the last two figures as the reviewer suggests. We have combined the electrophysiology data for 4 and 8 sec into one figure (Figure 5, see lines 460-561) and split out the reaction time data into a separate figure (Figure 6, see lines 562-580). In addition, we have made several stylistic and statistical changes we hope all enhance clarity: • The 4-sec and 8-sec PCA analysis is now side by side in Figure 5 for easier comparison and have rewritten the results such that the statistics better accompany the flow of the figures and the comparisons made between the 4 and 8-sec delay data (see lines 460-581). • We have reworked the Key (Figure 5, top) for interpreting the trajectories and hope that this will aid in interpretation of the PCA trajectory data. To this point, we have also provided definitions throughout several points of the document as to what the trajectories represent to assist in interpretation (see lines 311-320, 462-464, 871-873 &Figure 5 key). • We have elected to remove the arrows from the PCA 3D graph (Figure 5A, B) in order to simplify the graphs and remove unnecessary visualizations that are not essential to the interpretation of the data. We simplified the PCA 3D graphs (Figure 5A, B) to include descriptors of task features encoded by the individual PCs. • We have chosen to keep the color combinations and dashed vs solid lines to explain what each of the trajectories represent within the individual PC plots (Figure 5C, D). However, we added additional descriptors to the titles to help interpret the trajectory data within each PC (Figure 5C, D). These descriptors within the titles (Figure 5C, D) complement the additional descriptors in the 3D PCA graphs (Figure5A, B) to make the transition more seamless. • We chose to combine the timeseries graph depicting the change in Euclidean distance over time with the average distance over time as an inset (Figure 5E, F, see lines 523-561). o Notably, we now directly compared the PCA distance metrics across delays and follow up this comparison by assessing distance metrics within each delay (repeated measures ANOVAs and Scheffe post-hoc tests; see lines 508-514, 516-522, 523-552) o At each delay, we also report differences between 'Change' and 'Fail-to-Change' at individual time points (FDR-corrected t-tests; see lines 554-561).

We think that the stylistic changes and additional statistical analyses enhance the clarity and flow of the document and are important for addressing our underlying hypotheses.

R1.6: The dorsomedial PFC is not the most common terminology applied to the region/s being studied, which appear to be prelimbic and potentially anterior cingulate cortex. I would encourage the authors to embrace these terms to allow their work to better connect to other studies which have examined these regions. The choice of "dmPFC" serves to obscure this information, without a clear rationale for doing so." We have updated the manuscript and used the terminology anterior cingulate cortex (ACC).

Reviewer #2 Rationale for Significance Rating for Authors: "The modified discounting paradigm takes into account potential reward utility that is monitored by the animals which is influenced by the activity of the mPFC. The major limitation is the interpretation of this behavior in terms of planning, exploration/exploitation." We thank the reviewer for their comments and address each below.

Comments to the authors: "White and colleagues assessed how inhibition of the dmPFC or dmPFC activity associated with delay discounting behavior. Animals were trained on a modified delay discounting task where the delayed, large reward was consistent (but delays increased between sessions) and the immediate reward varied between trials based on previous behavior. Behavior was analyzed for discounting as well as "planning" based on the latency to initiate a trial and the latency to make a choice. Animals exhibited lower mean ranks for latencies for delay trials regardless of the immediate reward value. The planning index was higher for delay choices, especially later in training for high immediate reward values. Inactivation of the dmPFC increased selection of the immediate reward at the 8 second delay which appeared to be associated with increased choice of the immediate reward when the i-value was low. mPFC cells become active to delivery of the reward, and in-depth PCA analysis showed mPFC differed at the 4 and 8 second delays based on "explore" or "exploit" strategies. The authors conclude that the inactivating the mPFC during deliberation increases impulsivity during the 8 second delay and that this is due to a shift towards "exploitation" of a disadvantageous strategy. The model is interesting, single unit recordings are novel, and the PCA strategy is interesting. However, there are some concerns mainly related to presentation and interpretation:

We thank the reviewer for their positive comments.

Major:

R2.1: In general the manuscript is incredibly dense and challenging to read, and while some portions are thoroughly described, others are unclear. The authors provide lengthy descriptions of exclusion criteria and selection of behavior, but then do not adequately describe their statistical approach (when ANOVA was selected, alpha value, etc.) or calculations of other metrics. For example, when calculating the planning index, does this take into account all delays or just the ones where discounting is observed? Do the planning metrics differ by delay? Clarifying each section along with clear presentation of task structure (including training days in a figure), description of analytic strategy, and results is recommended.

We have taken several steps to enhance clarity of the analysis and statistical approaches used. We have rewritten several parts of the methods and results to enhance clarity. In addition, we have made several changes to the figures to make the statistical approach more intuitive. • Similar issues regarding clarity were raised by Reviewer 1. Please see our responses to R1.3-5 (above) for a description of the changes to the manuscript.

In addition, in response to the specific points raised in R2.1 please see the changes described below. • We have clearly stated what statistical analyses were used throughout the documents methods and results and included a sentence stating that all significance a-values were set at 0.05 (see lines 291-292). • We have rewritten parts of the methods describing the individual analyses, with special attention paid to the PCA analyses and distance calculations (see lines 321-352). • We added analyses of immediate/delay high/low i-value firing rates (Figure 4B, C; see lines 300-308) to follow-up on the finding from the optogenetic study where we report a greater number of consecutive immediate low value rewards for Laser ON sessions (Figure 3A).

R2.2) In contrast to traditional delay discounting paradigms where the immediate reward is held constant, the utility of the reward seems to change based on the i value and delay. However, this is unclear which affects interpretation. Rats select an equal number of immediate and delayed choices at the 8 second delay and discount at 16 seconds. Given that 8-16 second delays would typically not impact discounting in a rat, the immediate reward must be incentivized at these short delays. The opto manipulation increases choice of the immediate reward and the authors describe this is as maladaptive impulsivity but it is unclear if this is the result intolerance to delay or a shift in behavior based on changes in utility of the reward. Do the laser-ON sessions result in fewer rewards? A figure showing number of pellets earned at the different delays (including with opto manipulation) is recommended.

Excellent suggestion. We have included a figure that shows that with activation of the laser that fewer pellets are earned at both the 8 and 16 sec delays (Figure 2C; see lines 397-416 for discussion of results) R2.3) It isn't clear from the data and task if the interpretation of behavior presented is truly "planning". Decreased latency could reflect planning, but could also reflect increased motor impulsivity or even perseveration.

We agree that more work is required to adequately capture behavioral and neural correlates of planning. Therefore, we have removed this from the manuscript entirely. See response R1.4.

R2.4) The behavior described as "exploitation" (responding for the same option even after forced choice) could have alternate explanations. Exploitation strategies typically become more robust and stable regardless of the immediate outcome (as the animal has a representation of the overall outcome/preference and doesn't need to explore to make the choice). The example data provided in figure 1B do not suggest a true "exploit" strategy, but rather, the animal selects the same option 2 times followed by one forced choice and then one additional selection of the same choice before switching. This doesn't suggest exploitation but perhaps inflexibility or possibly mild perseverative responding.

We agree with the reviewers point. We have removed all references to exploitation and now refer to three or more of the same consecutive choices as "runs". As suggested, we do not reach the bar for proving this is an exploiting behavior and doing so is not necessary to support the main finding of the paper.

We have provided a definition of a "run" in the methods section (see lines 311-320) and use and reinforce this terminology throughout the results and discussion. We also give an example of 'runs' in Figure 1B. This terminology is also utilized in Figures 5 and 6, as we use the last trials of a run in our PCA analyses (Figure 5) and choice latency analyses (Figure 6). Our definition of a "run" is as follows, Runs were defined as three or more consecutive choices on the same lever followed by either continuing the run on the 4th trial (Fail-to-Change run) or changing levers and ending the run on the 4th trial (Change run) for the immediate and delay levers. We reinforce this definition at points in the text where we discuss analyses or results of runs (311-320, 462-464, 873-875 &Figure 5 key).

R2.5) The authors oscillate between describing their manipulation as the PL or dmPFC. Although expression of the virus is presented for the opto experiments the authors do not present placement of the optic fibers, so it is not possible to determine if it is just PL or dmPFC undergoing opto manipulations. The authors also state that they confirm glutamatergic specificity of the virus, but do not specify which antibody/ morphological criteria was used to do this. No representative image of the electrode placement is provided.

We have included optic fiber placements in Figure 3A and the electrode placements in Figure 4A. In the prior version of the manuscript, we specified PL for the opto results because the end of the optic fiber is positioned right above the PL, therefore even if there is viral expression in the ACC, the light is not directly targeting those cells due to the position of the optic fiber. In contrast, most of our ephys data was conducted using electrodes on moveable drives, therefore, the neural recordings (especially the early sessions) could have had ACC as well as PL (dmPFC). However, we realize now that our efforts to use precise terminology made the manuscript more confusing (this exact point was raised by R1). Therefore, we now refer to ACC throughout.

Minor:

1) Results are presented out of order (figure 3, then 5, then 4).

We have updated the order of the results.

2) There are several typos, missing components, and mislabeled features throughout the manuscript (e.g. Figure 3C the y-axis is i-value but seems like it should be indifference point).

Thank you for pointing out the inconsistency. Please note that we have moved the referenced figure (Figure 3C) to Figure 1C in the current document revision. We have changed the y-axis to 'indifference point' (Figure 1C).

3) Individual data points are not provided for most of figure 1." We have included individual points for figure 1.
